# Carboxypeptidase N2 as a Novel Diagnostic and Prognostic Biomarker for Lung Adenocarcinoma

**DOI:** 10.3389/fonc.2022.843325

**Published:** 2022-05-23

**Authors:** Ting Xu, Zhe Zhang, Hongqiang Chen, Ruili Cai, Qian Yang, Qi Liu, Yahan Fan, Wenbin Liu, Chunyan Yao

**Affiliations:** ^1^Department of Blood Transfusion, Southwest Hospital, Third Military Medical University (Army Medical University), Chongqing, China; ^2^Department of Breast and Thyroid Surgery, Daping Hospital, Third Military Medical University (Army Medical University), Chongqing, China; ^3^Department of Environmental Health, College of Preventive Medicine, Third Military Medical University (Army Medical University), Chongqing, China; ^4^Institute of Toxicology, College of Preventive Medicine, Third Military Medical University (Army Medical University), Chongqing, China; ^5^Institute of Pathology and Southwest Cancer Center, Southwest Hospital, Third Military Medical University (Army Medical University), Chongqing, China; ^6^Key Laboratory of Tumor Immunopathology, Ministry of Education of China, Chongqing, China

**Keywords:** *CPN2*, lung adenocarcinoma, biomarker, diagnosis, prognosis

## Abstract

Carboxypeptidase N2 (CPN2) is a plasma metallo-protease that cleaves basic amino acids from the C-terminal of peptides and proteins. Emerging evidence showed that carboxypeptidases perform many diverse functions in the body and play key roles in tumorigenesis. However, the clinical significance and biological functions of CPN2 in lung adenocarcinoma remain unclear. Our study aimed to explore the potential role and functions of CPN2 in lung adenocarcinoma. The results showed that the transcription level of *CPN2* was significantly increased in the tumor tissues of lung adenocarcinoma patients compared to the adjacent normal tissues in The Cancer Genome Atlas cohort (*P* < 0.05). The survival plots showed that the overall survival of patients with a high expression of *CPN2* was significantly lower than that of patients with a low expression of *CPN2*, both in the Kaplan–Meier database and the clinical sample cohort (*P* < 0.05). The tissue microarray analysis found that CPN2 protein expression was significantly positively correlated with node status and tumor stage as well as tumor malignancy (*P* < 0.05). Further univariate and multivariate Cox regression analyses showed that CPN2 may act as an independent prognostic factor in patients with lung adenocarcinoma (*P* < 0.05). In addition, the analysis of co-expression genes from LinkedOmics showed that *CPN2* was positively associated with many genes of fibrillar collagen family members and the PI3K-Akt pathway. The gene set enrichment analysis showed that a higher expression of *CPN2* may participate in mTOR, TGF-BETA, NOTCH, TOLL-like-receptor, WNT, and MAPK signaling pathway in lung adenocarcinoma. Notably, the knockdown of *CPN2* significantly inhibited the ability of cell proliferation, clone formation, invasion, and migration. Our findings suggested that the upregulation of CPN2 is associated with a worse clinical outcome in lung adenocarcinoma and cancer-related pathways, which laid the foundation for further research on CPN2 during carcinogenesis.

## Introduction

Lung cancer is the most common cancer with the highest incidence and mortality rate among human tumor diseases worldwide. According to pathology, lung cancer can be divided into small cell lung cancer (SCLC) and non-small cell lung cancer (NSCLC), in which NSCLC accounts for 85% of the total number of lung cancer. Lung adenocarcinoma (LUAD) is the main type of NSCLC, accounting for about 40%, and its incidence and mortality are increasing (1). Effective early diagnosis and screening have always been the focus of cancer prevention and treatment of LUAD. Research showed that effective screening of high-risk populations can improve the survival rate of LUAD patients by 10–50 times (2). At present, the diagnosis of LUAD mainly depends on pathological analysis and image diagnosis, which have some limitations. Some diagnostic markers have been widely used in the clinical diagnosis of lung cancer, such as *CYFRA 21-1*. However, the diagnostic rate of *CYFRA 21-1* is low in LUAD and SCLC (3). Therefore, finding and identifying novel sensitive and specific tumor biomarkers is the key problem in the clinical research of lung cancer.

Carboxypeptidase (CP), including *CPN2*, *CPH/E*, *CPA*, *CPB*, *etc* is a type of zinc finger-like metalloproteinases in plasma. It has the activity of catalyzing the hydrolysis of carboxyl terminal amino acids in polypeptide chain, which is closely related to many important biochemical reactions in the body (4–6). Increasing evidence showed that *CP* is closely related to the occurrence, development, and prognosis of a variety of diseases and has an important clinical application value in the early diagnosis and prognosis evaluation of tumors (7–9). It has been reported that *CPE* could inhibit the migration and invasion of fibrosarcoma cells or upregulate the expression of *Bcl-2* by activating the ERK1/2 pathway to promote the proliferation of hepatocarcinoma cells (10). The expression of *CPE* was related to the recurrence survival rate and pathological stage of liver cancer, suggesting that the expression level of *CPE* could predict the prognosis of tumor (11).

*CPN2* plays a vital role in the process of regulating vasoactive peptide hormones, growth factors, and cytokines by specifically cleaving their C-terminal basic residues. In recent years, emerging evidence suggested that *CPN2* performs a crucial biological function in the invasion and migration of breast cancer and can be used as a biomarker for effective diagnosis and treatment of breast cancer (12, 13). However, up to now, there has not been any research on the role of *CPN2* in the early diagnosis of LUAD and its molecular mechanism.

In the present study, we found that the upregulation of transcription and the translation levels of CPN2 were significantly associated with worse survival outcomes in LUAD patients. Our results suggested that CPN2 may be an effective diagnostic and prognostic marker and play an important physiological role in the progression of LUAD. It provides a novel tumor biomarker for diagnosis, therapeutic, and prognostic purposes among LUAD patients.

## Materials and Methods

### The Cancer Genome Atlas Data Collection and Processing

The Cancer Genome Atlas (TCGA) dataset was employed to analyze the *CPN2* transcription level and the relationship between *CPN2* with the clinical characteristics. The level 3 RNA-seq data of 109 normal samples and 1,015 lung cancer samples were downloaded from UCSC website (https://xenabrowser.net/). The gene transcription level was estimated as log_2_ transformed reads per kilobase per million mapped reads normalized count.

### Kaplan–Meier Plotter Analysis

The Kaplan–Meier plotter (www.kmplot.com) was capable of assessing the prognostic effect of 54,000 genes across 21 cancer types. The portal includes gene chip and RNA-seq data source from Gene Expression Omnibus (GEO), TCGA, and European Genome–phenome Archive (EGA). The Kaplan–Meier plotter database was used to analyze the association between *CPN2* expression by the validated probe (216223_at) and overall survival (OS) among lung cancer patients. The median cutoff value of *CPN2* expression equal to 24 was employed to separate the patients into the high-expression group and low-expression group in each cohort. *P <*0.05 was regarded as statistically significant.

### Analysis of Co-expression Genes

Co-expression genes about *CPN2* (|Pearson coefficient| >0.3) in TCGA— (LUAD) were gathered from LinkedOmics database (http://www.linkedomics.org/) and visualized in Cytoscape 3.7.2. The unrelated genes in this network were removed, and degree scores were calculated through cytoHubba algorithm. The top 10 genes were considered the most related genes and shown in yellow dots. The corresponding Kyoto Encyclopedia of Genes and Genomes (KEGG) enrichment about these co-expression genes was also presented by R project (4.0.2).

### Protein–Protein Interaction Analysis

The protein–protein interaction analysis of CPN2 was conducted by the Search Tool for the Retrieval of Interacting Genes/Proteins (STRING) database (http://string-db.org/) using default parameters. Gene Ontology (GO) and KEGG analyses were performed by R project (4.0.2). The top 10 results are shown in a bubble plot, and the adjusted *P* value <0.05 was considered statistically significant.

### Gene Set Enrichment Analysis

The gene set enrichment analysis (GSEA; https://www.gsea-msigdb.org/) was conducted by GSEA software, version 4.0.3. The gene matrix of LUAD in TCGA was separated into two groups depending on *CPN2* expression, and different pathways were performed by this analysis. False discovery rate (FDR) <0.05 was considered statistically significant. The top 10 enriched pathways were shown by R project (4.0.2).

### Immunohistochemistry

The tissue microarray obtained from Shanghai Outdo Biotech Co., Ltd. of China contains 94 LUAD samples and 86 adjacent normal samples, which undergo surgical resection between September 2004 and April 2009. The follow-up time ranged from 1 month to 10 years. The use of clinical specimens for research purposes has been approved by the Research Ethics Committee of Shanghai Outdo Biotech Co., Ltd. The criteria for inclusion were as follows: diagnosed with LUAD by pathological method and has complete survival information. All clinical specimens were treated with conventional methods, and immunohistochemistry was performed to detect the expression of CPN2 protein through previously described protocols (14). About 4-μm-thickness sections were incubated with the anti-CPN2 antibody. The immunohistochemical results were read and interpreted independently by two senior pathologists who were blinded to the sample information. Tumor cells with partial or complete membrane staining were positive at any intensity. According to the ratio of chromogenic cells, the scoring criteria were as follows: 0: negative, 1: positive <25%, 2: positive at 25–49%, 3: positive at 50–74%, and 4: positive at 75–100%. According to the staining degree of cells, no staining was 0, light yellow was 1, brownish yellow was 2, and brown was 3. The immunohistochemical score was obtained by multiplying the two indexes (intensity of staining and number of positively staining cells). The expression of CPN2 was considered to be high expression if the multiplication score was more than 6.

### Cell Lines and Culture

The human lung cancer-derived cell line A549 was obtained from the American Type Culture Collection (Manassas, VA, USA). The cell line was recently authenticated and tested for mycoplasma contamination. The cells were routinely cultured in Dulbecco’s modified Eagle’s medium supplemented with 10% fetal bovine serum in an incubator with a humidified atmosphere of 5% CO_2_ at 37°C.

### RNA Extraction and Quantitative Reverse Transcription–Polymerase Chain Reaction Analysis

Total RNA was isolated from cells using TRIzol reagent (Invitrogen, USA) according to the manufacturer’s protocol. The cDNA was synthesized from 2 μg total RNA with PrimeScript^®^ RT Reagent Kit with gDNA Eraser (Takara, Japan). The mRNA expression was measured through quantitative reverse transcription–polymerase chain reaction (qRT-PCR) by using SYBR Premix Ex Taq (Takara, Japan). The primer sequences are shown in **Supplementary Table S1**. The relative expression levels among the different samples were calculated using the 2^-ΔΔCt^ method with normalization to actin. The experiments were performed at least three times.

### Cell Transfection

For *CPN2* knockdown, the control siRNA and knockdown siRNA of *CPN2* gene were synthesized by Jima (Shanghai, China). The target sequences of *CPN2* gene knockdown that were used for siRNA are listed in **Supplementary Table S2**. For *CPN2* overexpression, the cDNA of *CPN2* gene was cloned into mammalian expression vector pcDNA3.1-T2A-EGFP. The cells were transiently transfected with those plasmids by ViaFect Transfection Reagent (Promega) according to the manufacturer’s protocol. After 48 h of transfection, the cells were used for subsequent functional experiments.

### Cell Proliferation

For the cell proliferation assay, lung cancer cells at a density of 5,000 cells per well were seeded into 96-well plates. After culturing for 24 h, the cells were transfected with the control siRNA and knockdown siRNA of *CPN2* gene. The optical density value was detected by measuring the absorbance at 450 nm after 1, 2, 3, and 4 days by using a CCK-8 kit (Dojindo, Japan) according to the manufacturer’s guidelines. All assays were carried out at least in triplicate.

### Colony Formation Assay

For the cell colony formation assay, after transfection for 24 h, about 500 lung cancer cells were seeded into 6-well plates and cultured. About 3 weeks later, the cell colonies (clones which contained >50 cells were included in the statistics) were stained with crystal violet (0.1%), counted, and photographed. All assays were carried out at least in triplicate.

### Cell Migration and Invasion Assays

For the wound healing assay, transfected cells were cultured in six-well plates until confluent. After scratching the monolayer, the cells were photographed at 0, and 48 h. Images were taken from five random optical fields on each filter. For the Transwell assay, a Transwell chamber (Corning, USA) which was coated with or without Matrigel mix (Corning, USA) was used to assess cell invasion and migration, respectively. After being transfected, 2 × 10^4^ cells were plated in the top chamber with serum-free medium, and a medium containing 10% fetal bovine serum was used in the lower chamber as a chemoattractant. After incubation for 24 h, the cells located on the bottom of the chamber were fixed with 4% paraformaldehyde for 15 min, stained with crystal violet (0.1%) for 15 min, and photographed under a microscope. The migrated or invaded cells were counted in five randomly selected fields in each well. Each sample was assayed in triplicate.

### Statistical Analysis

SPSS 20.0 software (SPSS, Inc., Chicago, IL, USA) was used to perform the statistical analyses. The difference in gene expression between normal and tumor tissues was analyzed using *t*-test. The survival plot was analyzed by the Kaplan-Meier method. Different clinical pathological factors and *CPN2* expression were considered single factors and performed through univariate and multivariate Cox regression. *P <*0.05 was considered statistically significant for all groups.

## Results

### *CPN2* Expression Increased Significantly in LUAD From the TCGA Cohort

**Figure 1:**
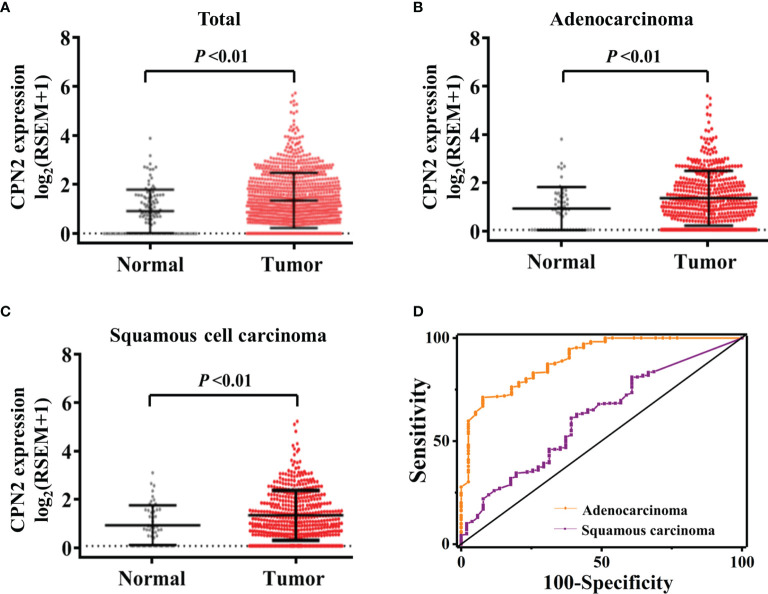
The *CPN2* transcription level was upregulated in lung cancer patients from The Cancer Genome Atlas cohort. **(A)** *CPN2* expression was significantly upregulated in lung cancer tissues compared with the adjacent normal samples according to the total samples (*P* < 0.01). Two-tailed Wilcoxon test. **(B, C)** *CPN2* expression was significantly increased in lung adenocarcinoma **(B)** and squamous cell carcinoma **(C)** tissues compared with the adjacent normal samples (*P* < 0.01). Two-tailed Wilcoxon test. **(D)** The receiver operating characteristic curve showed that the *CPN2* gene expression level was a promising biomarker with high sensitivity and specificity for the clinical diagnosis of lung adenocarcinoma, but not squamous cell carcinoma.

To assess the impact of *CPN2* expression on lung cancer, we assembled gene expression datasets from TCGA cohort. *CPN2* expression was significantly upregulated in lung cancer tissues compared with adjacent normal tissues (*P* < 0.01; **Figure 1A**). Then, we divided all lung cancer samples into two groups: adenocarcinoma and squamous cell carcinoma (LUSC). We found that *CPN2* expression was increased both in LUAD (*P* < 0.01; **Figure 1B**) and LUSC (*P* < 0.01; **Figure 1C**). In LUAD patients, *CPN2* expression was significantly upregulated in tumor tissues both in paired and unpaired samples compared with adjacent normal tissues (*P* < 0.01; **Supplementary Figure S1**).

In order to evaluate the diagnostic efficacy of *CPN2* in lung cancer patients, the receiver operating characteristic (ROC) curve was used to test the hypothesis. Compared with pathological analysis, the area under ROC curve (AUC) of *CPN2* gene expression in TCGA database for diagnosing LUAD was 0.86 (95% CI: 0.78–0.92), the sensitivity was 80.2%, and the specificity was 78.9%. The AUC of *CPN2* for diagnosing LUSC was 0.62 (95% CI: 0.54–0.70), the sensitivity was 62.9%, and the specificity was 85.25% (**Figure 1D**). It suggested that *CPN2* gene expression was a promising candidate biomarker of LUAD, which has potential clinical diagnosis prospect and application value.

### Association Between *CPN2* and Overall Survival in LUAD Patients From Public Database

**Figure 2:**
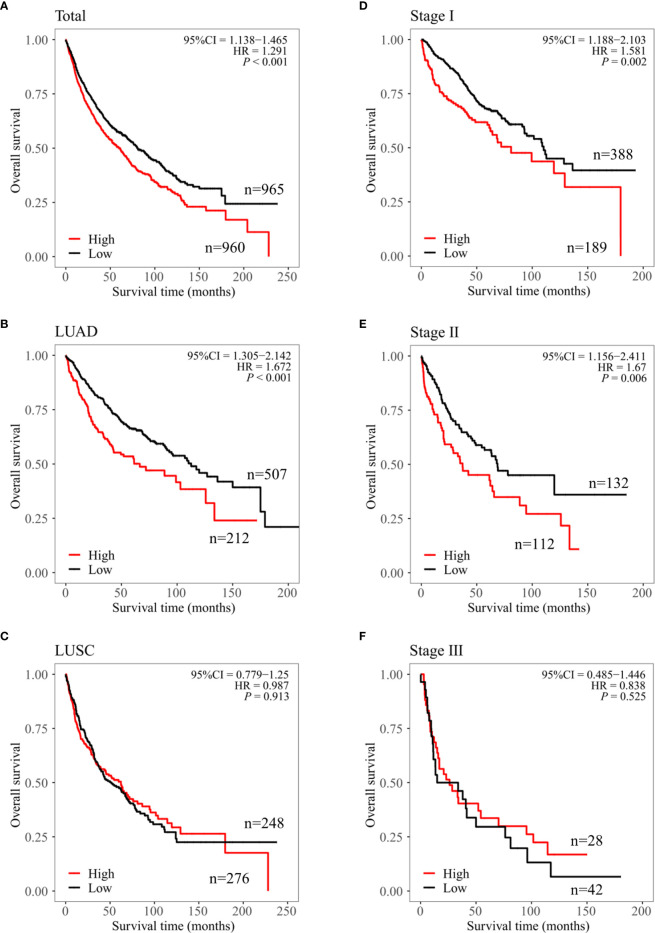
*CPN2* was associated with survival outcome in lung adenocarcinoma cancer from the Kaplan–Meier plotter. **(A–C)** Overall survival of *CPN2* in the lung cancer **(A)**, lung adenocarcinoma (**B**), and lung squamous cell carcinoma **(C)** cohorts, respectively. **(D–F)** Overall survival plot of *CPN2* in stage I **(D)**, stage II **(E)**, and stage III **(F)** in the lung adenocarcinoma cohort, respectively. Each group was divided according to the median expression cutoff value of CPN2 which equal to 24, and *P <*0.05 was considered statistically significant.

To evaluate the clinical significance of *CPN2*, we used the Kaplan–Meier public database to analyze the relationship between *CPN2* expression and the clinical outcomes of lung cancer patients. Survival plot showed that a higher *CPN2* was associated with poor overall survival (*P* < 0.001, **Figure 2A**). The subgroup analysis showed that a higher *CPN2* predicted a worse survival outcome in the adenocarcinoma group (*P* < 0.001, **Figure 2B**), but not in the squamous cell carcinoma group (*P* > 0.05, **Figure 2C**). The results showed that the expression level of *CPN2* gene was significantly related to the prognosis of LUAD. In patients with stages I and II, the OS time of patients with a low expression of *CPN2* gene was significantly longer than that of patients with a high expression of *CPN2* gene (*P* < 0.01) (**Figures 2D, E****)**. In patients with stage III, there was no significant correlation between the level of *CPN2* gene expression and the OS time of patients (*P >* 0.05) (**Figure 2F**). It suggested that *CPN2* can be used as a potential tumor marker for the prognosis of lung adenocarcinoma in early stage.

### High CPN2 Expression Was Associated With Poor Outcome of LUAD Patients

**Figure 3:**
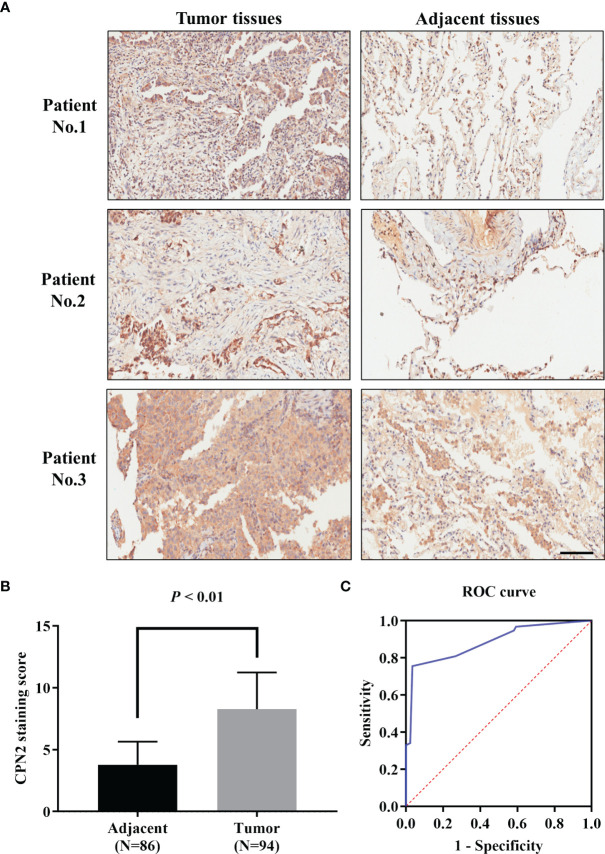
CPN2 expression was significantly upregulated in clinical lung adenocarcinoma tissues. **(A)** Immunohistochemistry analysis of CPN2 expression levels in adjacent and lung adenocarcinoma tissues, with the scale bar equal to 100 μm. CPN2 was highly expressed in tumor tissues. Immunohistochemistry staining assays were performed with an anti-CPN2 antibody (magnification, ×200). **(B)** CPN2 expression was significantly upregulated in lung adenocarcinoma tissues according to the total samples with a staining score. Two-tailed Wilcoxon test. **(C)** Receiver operating characteristic curve of CPN2 protein expression in clinical samples for the diagnosis of lung adenocarcinoma.

To further clarify the clinical significance of CPN2, immunohistochemical analysis was conducted in a tissue microarray of 94 LUAD tissues and 86 adjacent normal tissues. The results showed that CPN2 was expressed at lower levels in adjacent normal tissues; on the contrary, CPN2 was expressed at higher levels in LUAD tissues (**Figure 3A**). Compared with adjacent normal tissues, the expression of CPN2 was significantly upregulated in both paired and unpaired (*P* < 0.01, **Figure 3B**) LUAD tissues. In order to evaluate the value of CPN2 protein expression in the diagnosis of lung cancer patients, the ROC curve was used to test the hypothesis. The AUC of CPN2 protein expression for diagnosing LUAD was 0.88 (95% CI: 0.83–0.93, **Figure 3C**). It suggested that CPN2 protein expression was a promising candidate biomarker for LUAD.

### CPN2 Protein Upregulation Associated With the Poor Survival of LUAD Patients

Then, we calculated the correlation between the expression of CPN2 and the clinical pathologic parameters in LUAD patients. By dividing the cohort into two groups through the cutoff value of CPN2 protein expression as mentioned above, we found that the CPN2 expression status was significantly associated with lymph node status (*P* = 0.006), pathological N stage (*P* = 0.011), and American Joint Committee on Cancer (AJCC) stage (*P* = 0.035), but not with age, gender, grade, tumor size, total lymph nodes, pathological T stage, or pathological M stage (**Table 1**).

**Table 1:** Clinical factor and CPN2 expression in lung cancer patients.

Clinicopathologic parameters	Number	CPN2 expression status		*χ*^2^	*P*-value
		Low	High		
**Total**	94	52	42		
**Age (year)**				0.008	0.929
<60	43	24	19		
≥60	51	28	23		
**Gender**				0.305	0.581
Male	53	28	25		
Female	41	24	17		
**Grade**				0.940	0.332
1 + 2	61	36	25		
3	31	15	16		
Unknown	2	1	1		
**Tumor size**				0.007	0.934
<5 cm	72	40	32		
≥5 cm	22	12	10		
**Total lymph nodes**				0.206	0.651
<10	52	28	24		
≥10	41	24	17		
Unknown	1	0	1		
**Lymph node status**				7.479	0.006**
Negative	42	30	12		
Positive	51	22	29		
Unknown	1	0	1		
T (primary tumor)				0.118	0.731
T1 + T2	70	38	32		
T3 + T4	24	14	10		
**N (regional lymph nodes)**				6.425	0.011*
N0	42	30	12		
N1–3	37	16	21		
NX	15	6	9		
**M (distant metastases)**				0.816	0.366
M0	93	51	42		
M1	1	1	0		
**TNM stage**				4.463	0.035*
I and II	50	33	17		
III and IV	43	19	24		
Unknown	1	0	1		

*P < 0.05; **P < 0.01.

**Figure 4:**
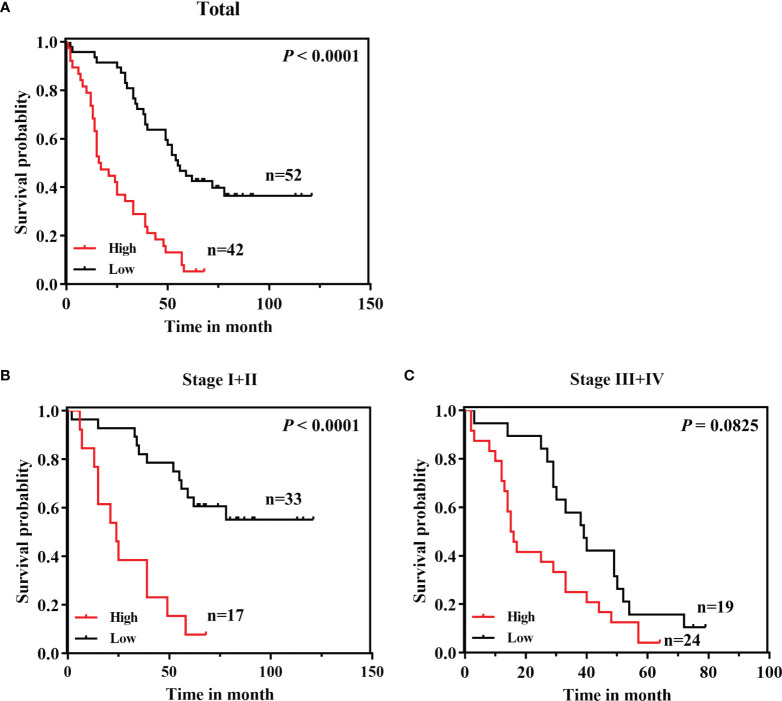
A high CPN2 protein expression was significantly associated with poor survival in clinical lung adenocarcinoma patients from our cohort by Kaplan–Meier survival curves. **(A)** The Kaplan–Meier survival curves based on a log-rank test showed that lung cancer patients with a high CPN2 expression had poorer survival than those with a low CPN2 expression (*P* < 0.0001). **(B)** Kaplan–Meier curves of lung adenocarcinoma patients with TNM stages I and II (*P* < 0.0001). **(C)** Kaplan–Meier curves of lung adenocarcinoma patients with TNM stages III and IV (*P* > 0.05).

To evaluate whether CPN2 protein expression correlated with the prognosis of LUAD patients, survival plots were addressed to determine OS in LUAD patients. The results showed that LUAD patients with a higher CPN2 expression had a significantly shorter OS than those with a lower CPN2 expression (*P* < 0.0001, **Figure 4A**). The subgroup analysis by AJCC stage revealed that this trend was significant only in stages I and II but not in stages III and IV (**Figures 4B, C****)**.

Furthermore, univariate and multivariate Cox regression were conducted to explore the prognostic value of CPN2 between multiple clinical characteristics. All samples separated by the median expression cutoff value of CPN2 were collected to perform this analysis. The results showed that upregulation of CPN2 significantly predicted a poor outcome in LUAD (HR = 3.753, 95% CI = 2.063–6.828, *P* = 0.001, **Table 2**). These results indicated that CPN2 possesses the potential capability to be an independent prognostic factor for LUAD.

**Table 2:** Univariate and multivariate Cox regression analyses of potential prognostic factors for lung cancer patients.

Clinicopathologic parameters	Univariate			Multivariate		
	HR	95% CI	*P*-value	HR	95% CI	*P*-value
Age (year) (<60 *vs*. ≥60)	0.953	0.582–1.562	0.849			
Gender (male *vs*. female)	0.759	0.465–1.239	0.270			
Grade (1–2 *vs*. 3–4)	1.022	0.607–1.719	0.936			
Tumor size (<5 cm *vs*. ≥5 cm)	1.735	0.983–3.062	0.057			
Total number of lymph nodes (<10 *vs*. ≥10)	1.310	0.799–2.148	0.284			
Positive number of lymph nodes (0 *vs*. ≥1)	2.648	1.534–4.572	<0.001***			
Tumor infiltration (T1–T2 *vs*. T3–T4)	1.422	0.830–2.434	0.200			
Lymph node metastasis (N0 *vs*. N1–3)	2.689	1.508–4.795	0.001**			
Distant metastasis (M0 *vs*. M1)	1.086	0.150–7.868	0.935			
TNM stag (I–II *vs*. III–IV)	2.502	1.498–4.177	<0.001***	2.408	1.339–4.330	0.003**
CPN2 expression (low *vs*. high)	3.661	2.187–6.129	<0.001***	3.753	2.063–6.828	<0.001***

HR, hazard ratio; CI, confidence interval.

**P < 0.01; ***P < 0.001.

### Analysis of Co-Expression Genes About *CPN2*

To further explore the meaningful function of *CPN2* in LUAD, co-expression genes were gained from LinkedOmics web tool. A total of 2,615 positively correlated genes (dark red dots) and 957 negatively correlated genes (dark green dots) were gathered and shown in a volcano plot (FDR < 0.05, **Supplementary Figure S2A**). The top 50 significant genes associated with *CPN2* were drawn in the heat map (**Supplementary Figures S2B, C****)**.

**Figure 5:**
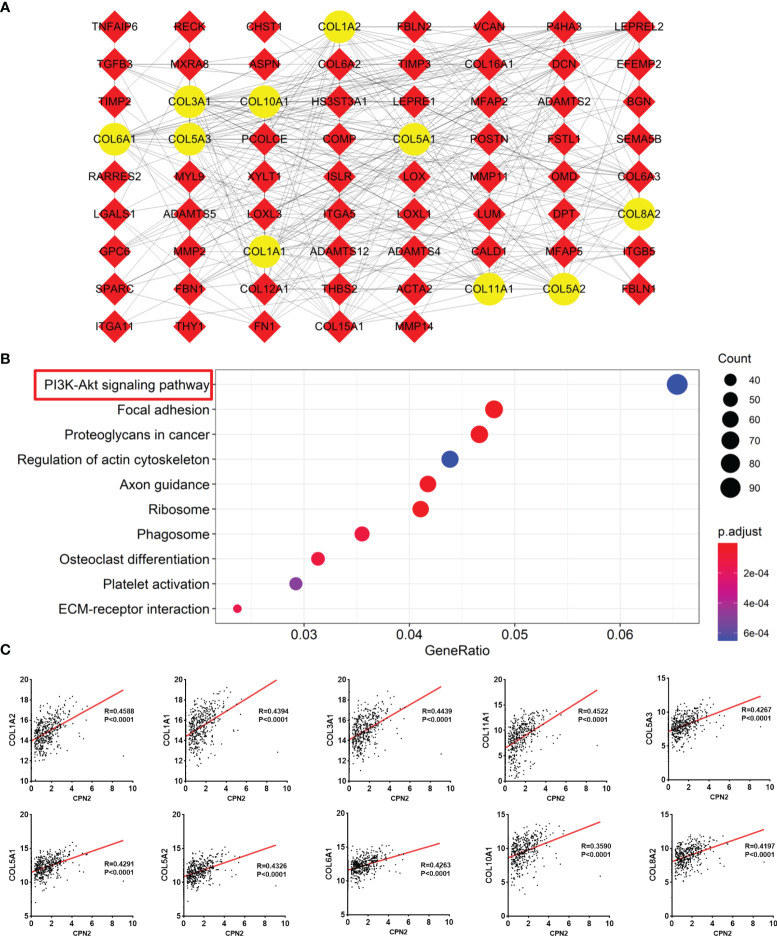
Co-expression genes of *CPN2* using Linkedomics analysis. **(A)** Protein–protein interaction of its co-expression genes. Co-expression genes with Pearson correlation coefficient >0.3 were gathered to draft this network, and the top 10 hub genes are shown in yellow color. **(B)** KEGG enrichment analysis of these co-expression genes. **(C)** Validation of 10 hub genes in The Cancer Genome Atlas lung adenocarcinoma database.

We gathered 242 co-expression genes using LinkedOmics analysis to speculate the similarity mechanism of *CPN2* in LUAD. The criteria were as follows: |Pearson coefficient| >0.3 and FDR < 0.05. The result is shown in **Figure 5A**. The top 10 hub genes were *COL1A2*, *COL1A1*, *COL3A1*, *COL11A1*, *COL5A3*, *COL5A1*, *COL5A2*, *COL6A1*, *COL10A1*, and *COL8A2*. Furthermore, the KEGG pathway enrichment analysis revealed the functional annotation of these genes. The results showed that the KEGG pathways were mainly focused on the PI3K-Akt signaling pathway, focal adhesion, proteoglycans in cancer, regulation of actin cytoskeleton, axon guidance, ribosome, phagosome, osteoclast differentiation, platelet activation, and extracellular matrix–receptor interaction (adjusted *P* < 0.05; **Figure 5B**). These enrichment results also validated the role of *CPN2* which acts as a potential oncogene in LUAD. In addition, these co-expression genes were validated in TCGA-LUAD database (**Figure 5C**) and presented significant correlations with *r* > 0.4.

### Protein–Protein Interaction and Functional Enrichment

**Figure 6:**
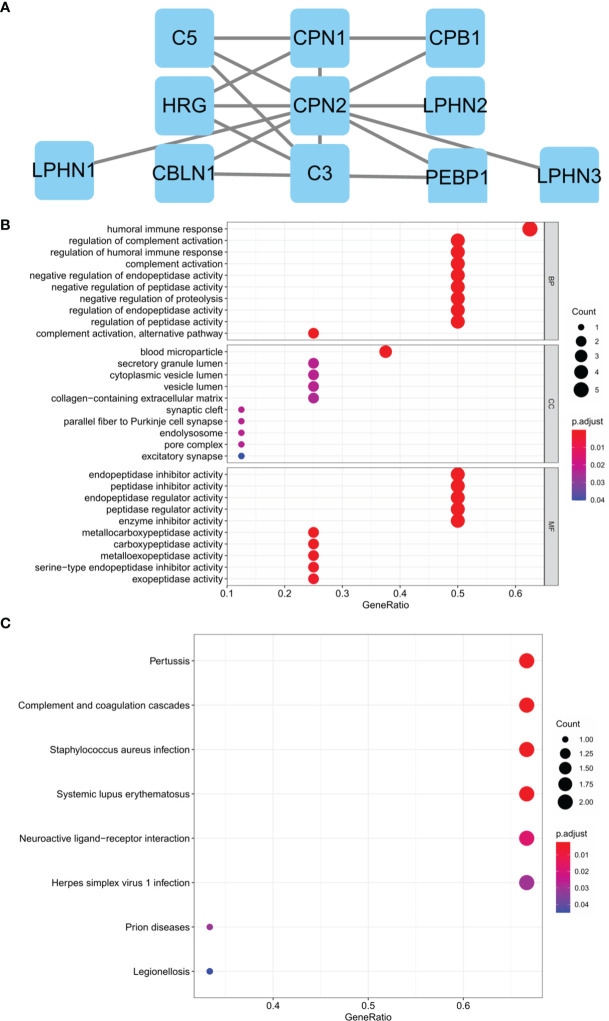
Protein–protein interaction network and functional enrichment analysis of genes interacted with *CPN2* through Search Tool for the Retrieval of Interacting Genes/Proteins (STRING) analysis. **(A)** Ten genes interacted with *CPN2* confirmed by STRING database. **(B)** Gene ontology biological process enrichment analyzed by these interaction genes. **(C)** Kyoto Encyclopedia of Genes and Genomes enrichment analyzed by these interaction genes. False discovery rate <0.5 was considered statistically significant.

Genes that interacted with *CPN2* were evaluated using STRING analysis and visualized by Cytoscape software. The 10 interaction genes were carboxypeptidase N, polypeptide 1 (*CPN1*), complement component 5 (*C5*), carboxypeptidase B1 (*CPB1*), histidine-rich glycoprotein (HRG), latrophilin 2 (*LPHN2*), latrophilin 1 (*LPHN1*), cerebellin 1 precursor (*CBLN1*), complement component 3 (*C3*), phosphatidylethanolamine binding protein 1 (*PEBP1*), and latrophilin 3 (*LPHN3*), and the interaction network is shown in **Figure 6A**. In addition, GO and KEGG enrichments of these interacted genes were also performed. The GO enrichment presented that regulation of complement activation, humoral immune response, regulation of humoral immune response, complement activation, negative regulation of endopeptidase activity, negative regulation of peptidase activity, negative regulation of proteolysis, regulation of endopeptidase activity, complement activation, alternative pathway, and regulation of peptidase activity were mainly enriched biological processes (**Figure 6B**). Consistently, pertussis, complement and coagulation cascades, *Staphylococcus aureus* infection, systemic lupus erythematosus, neuroactive ligand–receptor interaction, herpes simplex virus 1 infection, prion diseases, and legionellosis were mainly enriched KEGG pathways (**Figure 6C**).

### Gene Set Enrichment Analysis About *CPN2*

**Figure 7:**
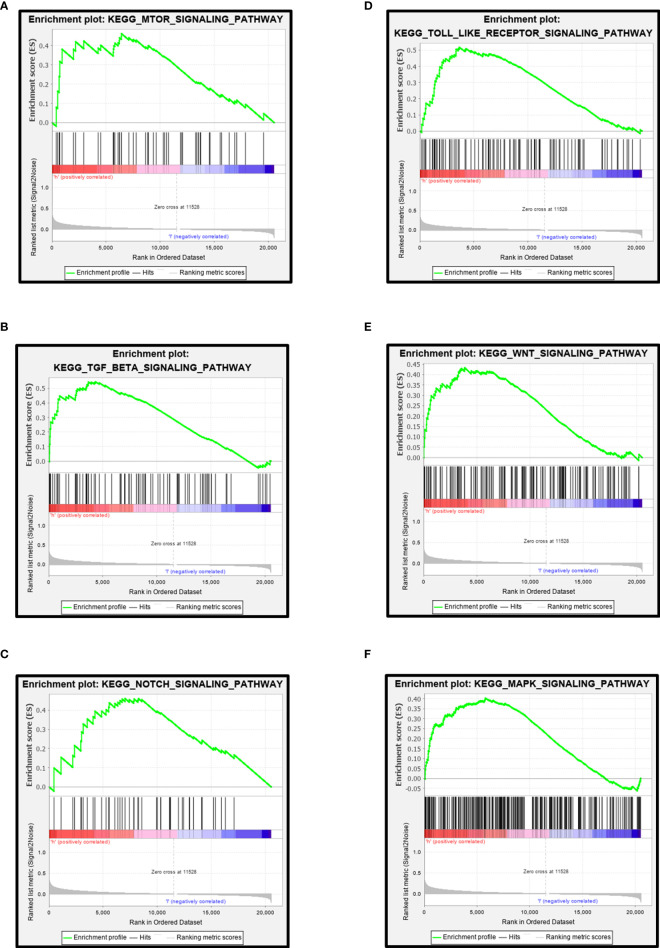
Gene set enrichment analysis of *CPN2* in lung adenocarcinoma. The high expression of *CPN2* was significantly enriched in the mTOR signaling pathway **(A)**, TGF-BETA signaling pathway **(B)**, NOTCH signaling pathway **(C)**, TOLL-like-receptor signaling pathway **(D)**, WNT signaling pathway **(E)**, and MAPK signaling pathway **(F)**. False discovery rate <0.25 was considered statistically significant.

GSEA was performed to further explore the functional role of *CPN2* in LUAD progression. We separated TCGA gene matrix into two groups based on *CPN2* expression and performed GSEA analysis. Many tumor-related pathways were significantly enriched between the two groups. From the results of the KEGG pathway analysis, we found that a high expression of *CPN2* was significantly enriched in the mTOR signaling pathway (**Figure 7A**), TGF-BETA signaling pathway (**Figure 7B**), NOTCH signaling pathway (**Figure 7C**), TOLL-like-receptor signaling pathway (**Figure 7D**), WNT signaling pathway (**Figure 7E**), and MAPK signaling pathway (**Figure 7F**). FDR <0.25 was considered statistically significant. Furthermore, the top 10 enriched pathways according to the normalized enrichment score are shown in **Supplementary Figure S3**. FDR <0.25 was considered statistically significant. Integrating the results of GSEA and co-expression gene enrichment, we propose that *CPN2* may play a vital role through cancer-related pathways.

### Knockdown of *CPN2* Inhibited Lung Cancer Cell Growth, Migration, and Invasion

**Figure 8:**
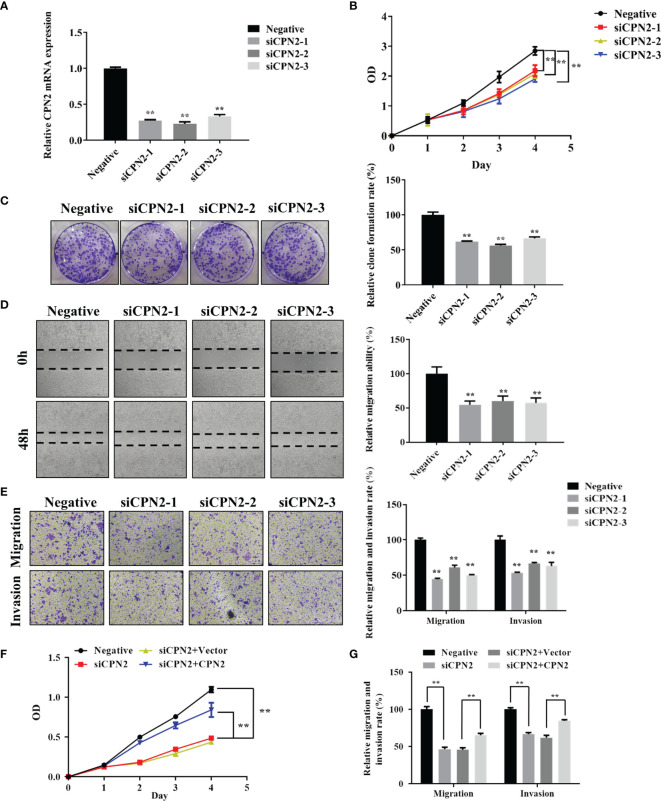
*CPN2* knockdown significantly inhibited lung cancer cell proliferation, migration, and invasion. **(A)** The knockdown expression of *CPN2* was confirmed by qRT-PCR in lung cancer cells. **(B)** CCK-8 assay was used to examine the effect of *CPN2* knockdown on proliferation in lung cancer cells. **(C)** Colony formation assays were used to examine the effect of *CPN2* knockdown on growth in lung cancer cells. **(D)** Wound healing assay was used to determine the motility of *CPN2* knockdown in lung cancer cells. The quantification of migrated cells is shown in the right panel. **(E)** Transwell assay was used to examine the effect of *CPN2* knockdown on migration and invasion in lung cancer cells. The quantification of migrated cells is shown in the right panel. **(F)** CCK-8 assay was used to detect the effect of *CPN2* overexpression on proliferation in lung cancer cells with *CPN2* knockdown. **(G)** Transwell assay was used to detect the effect of *CPN2* overexpression on migration and invasion in lung cancer cells with *CPN2* knockdown. All data represent the mean ± SD of three independent experiments. **P < 0.01.

For the cellular functional experiments, the loss of function assay was performed using *CPN2* silencing in lung cancer cell lines. Firstly, we knocked down the *CPN2* expression using siRNA vector in the A549 cell line used in our study. As shown by qRT-PCR, *CPN2* expression was significantly reduced after the transfection (**Figure 8A**). Then, the CCK-8 assay results showed that *CPN2* knockdown remarkably suppressed the proliferative ability of A549 cells (**Figure 8B**). The colony formation assay showed that the *CPN2* knockdown dramatically inhibited the clones’ number of lung cancer cells (**Figure 8C**). Then, the wound healing assay was used to detect the effect of *CPN2* knockdown on cell migration ability. As shown in **Figure 8D**, compared with the control group, the migration distance of cells was significantly reduced after the knockdown of *CPN2* (*P* < 0.01). At the same time, a Transwell assay was used to detect the migration and invasion ability of cells after *CPN2* knockdown. Transwell migration and Matrigel invasion assays showed that *CPN2* downregulation significantly inhibited the migratory and invasive capabilities of lung cancer cells, respectively (**Figure 8E**). In order to solidly establish the role of *CPN2* in lung cancer, we performed the rescue experiment with *CPN2* cDNA after the siRNA transfections to rescue the phenotype. The CCK-8 assay (**Figure 8F**) and Transwell assay (**Figure 8G** and **Supplementary Figure S4**) results showed that *CPN2* overexpression significantly promoted the cell growth, migration, and invasion ability after *CPN2* knockdown. In conclusion, these above-mentioned findings supported the conclusion that *CPN2* exerts an oncogenic role in lung cancer cells.

## Discussion

Carboxypeptidase is an enzyme that hydrolyzes peptides and especially polypeptides by sequentially splitting off the amino acids at the end of the peptide chain which contains free carboxyl groups. Many carboxypeptidases serve as material transport channel related to molecular biological process in the body and play key roles in major biological processes (6). An increasing number of studies indicated that carboxypeptidases have potentially vital functions in cancer research (7, 9, 10). Therefore, we explored the association between *CPN2* expression and clinical outcomes. Our study provided ample evidence for the importance of *CPN2* in carcinogenic properties and potential prognostic biomarker for LUAD. We found, for the first time, that the upregulated transcription and protein level of *CPN2* could be explored as a novel diagnostic and independent prognostic biomarker for LUAD. Besides this, *CPN2* could participate in some cancer-related pathways.

Carboxypeptidase N is important in the regulation of peptides, like kinins and anaphylatoxins, and has also been known as kininase-1 and anaphylatoxin inactivator. It has been reported that CPN can be used as a biomarker for the effective diagnosis and treatment of breast cancer (13). The serum and mRNA expression levels of CPA4 were also found to be prognostic biomarkers for breast cancer patients (15). In our study, the expression level of *CPN2* gene is a sensitive and specific biomarker for the clinical diagnosis of LUAD. Importantly, the high expression of *CPN2* showed worse survival only in stages I and II, but not in stages III, suggesting that *CPN2* expression is an independent prognostic biomarker for lung cancer in an early stage. However, our finding needs to be further confirmed in other groups with more populations. Other diagnostic methods such as chest radiographs and computed tomography are more convenient and effective for the diagnosis of lung diseases (16, 17). Recently, the combination of IgA and IgG autoantibodies against transcriptional intermediary factor-1γ was found to be useful for the early diagnosis of lung cancer (18). Interestingly, machine learning of seven serum metabolites and relevant pathways can be used as a biomarker panel for distinguishing early-stage LUAD from controls (19). It suggested that conventional examination combined with molecular biomarker analysis is a more valuable approach for the early diagnosis of lung cancer. However, this needs to be investigated in future studies.

In recent years, more and more carboxypeptidases have been found to be associated with tumorigenesis. The *CPA1* and *CPB1* variants induced by ER stress are associated with pancreatic cancer development (20). The coding variation in *CPA4* may confer an increased risk of intermediate-to-high risk prostate cancer among younger patients (21). Recent research suggested that *CPA4* plays an important role during the process of tumor microenvironment formation and distant metastasis. *CPA4* expression inhibited the tumor proliferation and regulated the expression of stem cell characteristics in hepatocellular carcinoma (22). Nevertheless, *CPA4* was found to be a key regulator of cardiac hypertrophy through activating PI3K-AKT-mTOR signaling and may serve as a promising therapy target for hypertrophic cardiac diseases (23). *CPA6* could promote cell proliferation and migration through regulating the AKT signaling pathway in hepatocellular carcinoma (24). It suggested that *CPA6* is a promising diagnostic biomarker and therapeutic target for hepatocarcinoma. Increased carboxypeptidase-D expression was associated with the upregulation of progression markers VEGF-C and Runx2 during breast cancer progression (25). Elevated carboxypeptidase-D played an anti-apoptotic activity in prostate cancer, which is inhibited by combined prolactin receptor and androgen receptor targeting (26). Recently, genome-wide CRISPR screening in 3D lung cancer spheroids found that the loss of carboxypeptidase D reduced tumor growth and its expression correlates with patient outcomes in lung cancer patients (27). *CPE* promotes the survival of cancer cells by upregulating the expression of anti-apoptotic protein *Bcl-2* and other pro-survival genes *via* the ERK1/2 pathway activation (10). Carboxypeptidase E-∆N promotes proliferation and invasion *via* the upregulation of *CXCR2* expression in pancreatic cancer (28). Carboxypeptidase E-ΔN promotes migration, invasion, and epithelial–mesenchymal transition *via* the Wnt/β-catenin pathway in human osteosarcoma (29). N-terminal-truncated carboxypeptidase E represses E-cadherin expression by stabilizing the Snail-HDAC complex in lung cancer (30). Carboxypeptidase X M14 family member 2 overexpression promotes proliferation and migration, predicts an unfavorable prognosis of osteosarcoma (31), and accelerates progression through the regulation of the gp130/JAK2/Stat1 pathway in hepatocellular carcinoma (32).

In our study, we found that a high CPN2 expression was associated with poor prognosis and is an independent prognostic biomarker in LUAD. It suggested that *CPN2* is closely related to the occurrence of LUAD. In order to further screen and find the key target of *CPN2* participating in the pathway of LUAD, we conducted a cluster analysis of the gene and signal pathway and found that the PI3K-Akt pathway was significantly associated with *CPN2* expression. Recent studies have shown that activated Akt mediates the growth, proliferation, and migration of tumor cells through the phosphorylation of downstream proteins (33, 34). It has been reported that the downstream molecules of the Akt pathway, such as *bad*, *caspase 9*, and *Bcl-2*, are inhibited under the action of activated Akt, thus losing the regulatory effect on cell apoptosis; *GSK-3* and *NF-κB* can promote cell proliferation and differentiation after Akt is activated (35). The results suggest that *CPN2* may be involved in the development of LUAD through the Akt pathway.

In order to further screen and find the downstream target of *CPN2* in the pathogenesis of LUAD, we used the protein interaction bioinformatics software and TCGA database analysis to find that the expression of *COL1A2*, *COL1A1*, and *COL3A1*, in the key target of the Akt pathway, was significantly positively correlated with the expression of *CPN2*. As recently reported, these genes belonged to the fibrillar collagen family members and showed a significant effect in tumor development from multiple aspects (36–38). From the results of the GSEA, we found that the high expression of *CPN2* was significantly enriched in the mTOR signaling pathway, TGF-BETA signaling pathway, NOTCH signaling pathway, TOLL-like-receptor signaling pathway, WNT signaling pathway, and MAPK signaling pathway. Our study found, for the first time, that *CPN2* acted as a novel oncogene and played an important role during the process of lung cancer. Combining the results of our study and related literature reports, we speculated that *CPN2* promoted tumor cell proliferation, invasion, and metastasis and inhibited apoptosis through Akt and the fibrillar collagen family or (and) this key pathway in the critical stage of LUAD. However, it should be clarified by further research in the future.

There are some limitations in this study. Firstly, parts of the data used in our study were publicly available. The difference between the number of normal samples and tumor samples is relatively large, which may lead to deviations due to the uneven number of samples. With the advancement of sequencing technology and the disclosure of more and more data, we can continue our study based on more samples. Secondly, the relevant results need to be verified on animal and clinical samples, and the subsequent experimental studies should focus on the mechanisms of *CPN2* carcinogenesis among LUAD patients.

## Conclusion

In summary, our study showed that *CPN2* expression level gradually increases with the increase of LUAD malignancy. *CPN2* has good detection performance as a diagnostic marker of LUAD. *CPN2* is an independent factor affecting the occurrence of LUAD, which is closely related to the prognosis of LUAD. Knockdown of *CPN2* significantly inhibited the ability of cell growth, invasion, and migration. The research results will provide new diagnostic markers with high sensitivity and high specificity for the early diagnosis of LUAD. It finally laid a solid theoretical foundation for the research and clinical promotion of novel non-invasive diagnostic methods of LUAD, yet further research and the underlying mechanism called for urgent exploration.

## Data Availability Statement

The datasets presented in this study can be found in online repositories. The names of the repository/repositories and accession number(s) can be found in the article/**Supplementary Material**.

## Ethics Statement

The studies involving human participants were reviewed and approved by the Research Ethics Committee of Shanghai Outdo Biotech Co., Ltd. The patients/participants provided their written informed consent to participate in this study.

## Author Contributions

WL and CY contributed to the conception of the study. TX, ZZ, and HC contributed to sample collection and data processing and performed the experiment. RC contributed significantly to analysis and manuscript preparation. QY participated in manuscript writing. QL and YF helped perform the analysis with constructive discussions. All authors contributed to the article and approved the submitted version.

## Funding

This work was supported by the National Natural Science Foundation of China (Nos. 81872659 and 82173556), the Natural Science Foundation Project of Chongqing CSTC of China (No. cstc2018jcyjAX0233), and the foundation of Youth Development Projects from the Southwest Hospital of The Third Military Medical University (SWH2018QNLC-08).

## Conflict of Interest

The authors declare that the research was conducted in the absence of any commercial or financial relationships that could be construed as a potential conflict of interest.

## Publisher’s Note

All claims expressed in this article are solely those of the authors and do not necessarily represent those of their affiliated organizations, or those of the publisher, the editors and the reviewers. Any product that may be evaluated in this article, or claim that may be made by its manufacturer, is not guaranteed or endorsed by the publisher.

## Supplementary Material

Supplementary Figure 1 *CPN2* transcription level upregulated in lung adenocarcinoma from The Cancer Genome Atlas (TCGA) cohort. **(A, B)** Scatter plot showing the expression of *CPN2* between tumor and normal tissues, tumor and adjacent normal tissues in TCGA database, according to unpaired **(A)** and paired **(B)** *t*-test, respectively. ***P* < 0.01, *****P* < 0.0001.

Supplementary Figure 2 *CPN2* co-expression genes in lung adenocarcinoma. **(A)** Volcano plot of *CPN2* co-expression genes according to Pearson coefficient. Red dots denote a positive correlation, and blue dots denote a negative correlation. **(B)** Heat map of the top 50 significantly positively correlated genes with *CPN2*. **(C)** Heat map of the top 50 significantly negatively correlated genes with *CPN2*.

Supplementary Figure 3 Kyoto Encyclopedia of Genes and Genomes enrichment analysis of its interacted genes through Search Tool for the Retrieval of Interacting Genes/Proteins analysis showing the top 10 enrichment pathways according to the normalized enrichment score. False discovery rate <0.05 was considered statistically significant.

Supplementary Figure 4 *CPN2* overexpression significantly promoted the migration and invasion ability in lung cancer cell with *CPN*2 knockdown by Transwell assay.

Supplementary Table 1 Primer sequences used for qRT-PCR.

Supplementary Table 2 Target sequences used for the knockdown of *CPN2*.
