# Supplementary figures and images for: Carboxypeptidase N2 as a Novel Diagnostic and Prognostic Biomarker for Lung Adenocarcinoma
Source: Front Oncol. 2022 May 23;12:843325. doi: 10.3389/fonc.2022.843325 (PMC9170673; doi:10.3389/fonc.2022.843325)

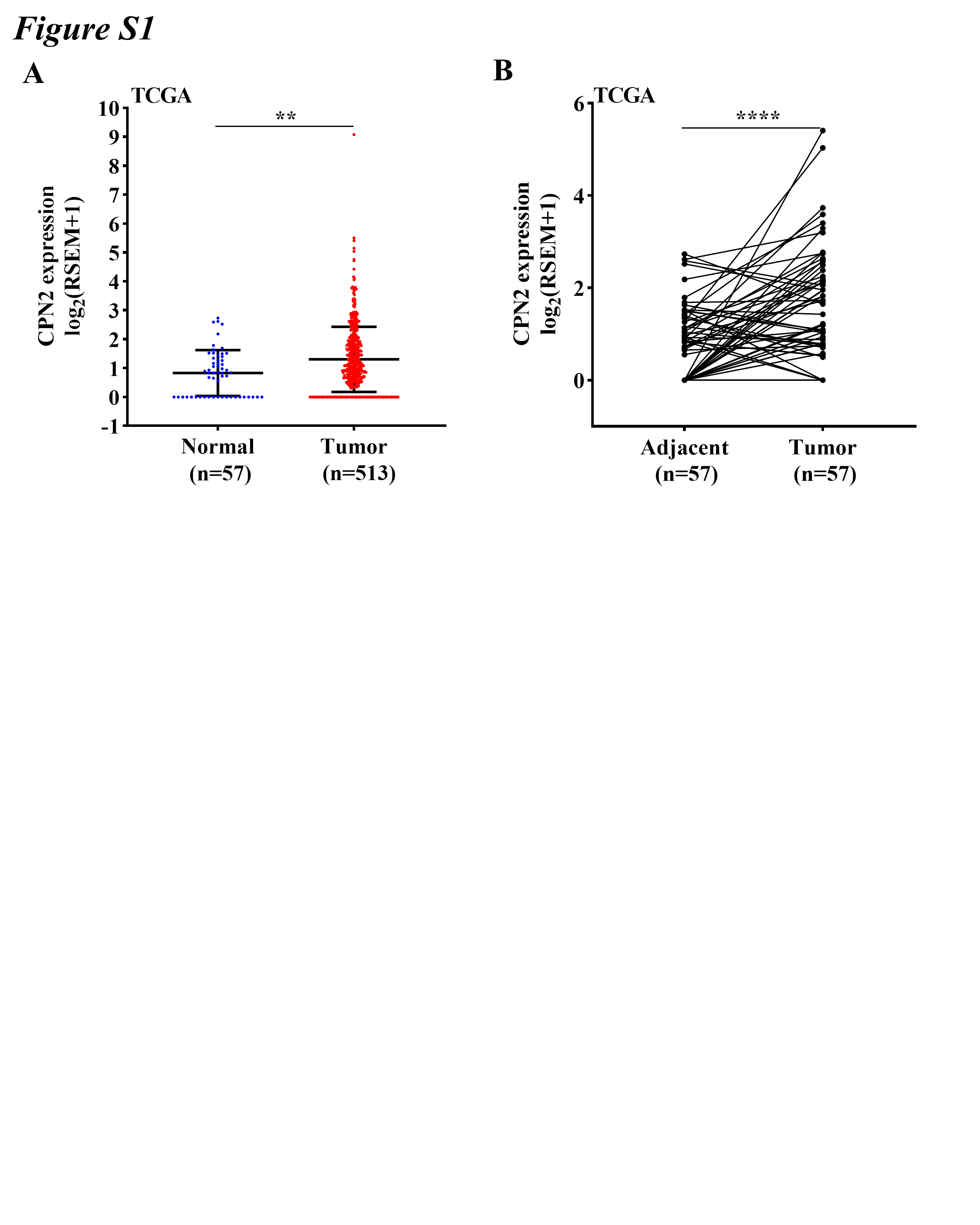

Supplement: Supplementary Figure 1 — CPN2 transcription level upregulated in lung adenocarcinoma from The Cancer Genome Atlas (TCGA) cohort. (A, B) Scatter plot showing the expression of CPN2 between tumor and normal tissues, tumor and adjacent normal tissues in TCGA database, according to unpaired (A) and paired (B) t-test, respectively. **P < 0.01, ****P < 0.0001. [file Image_1.tif]

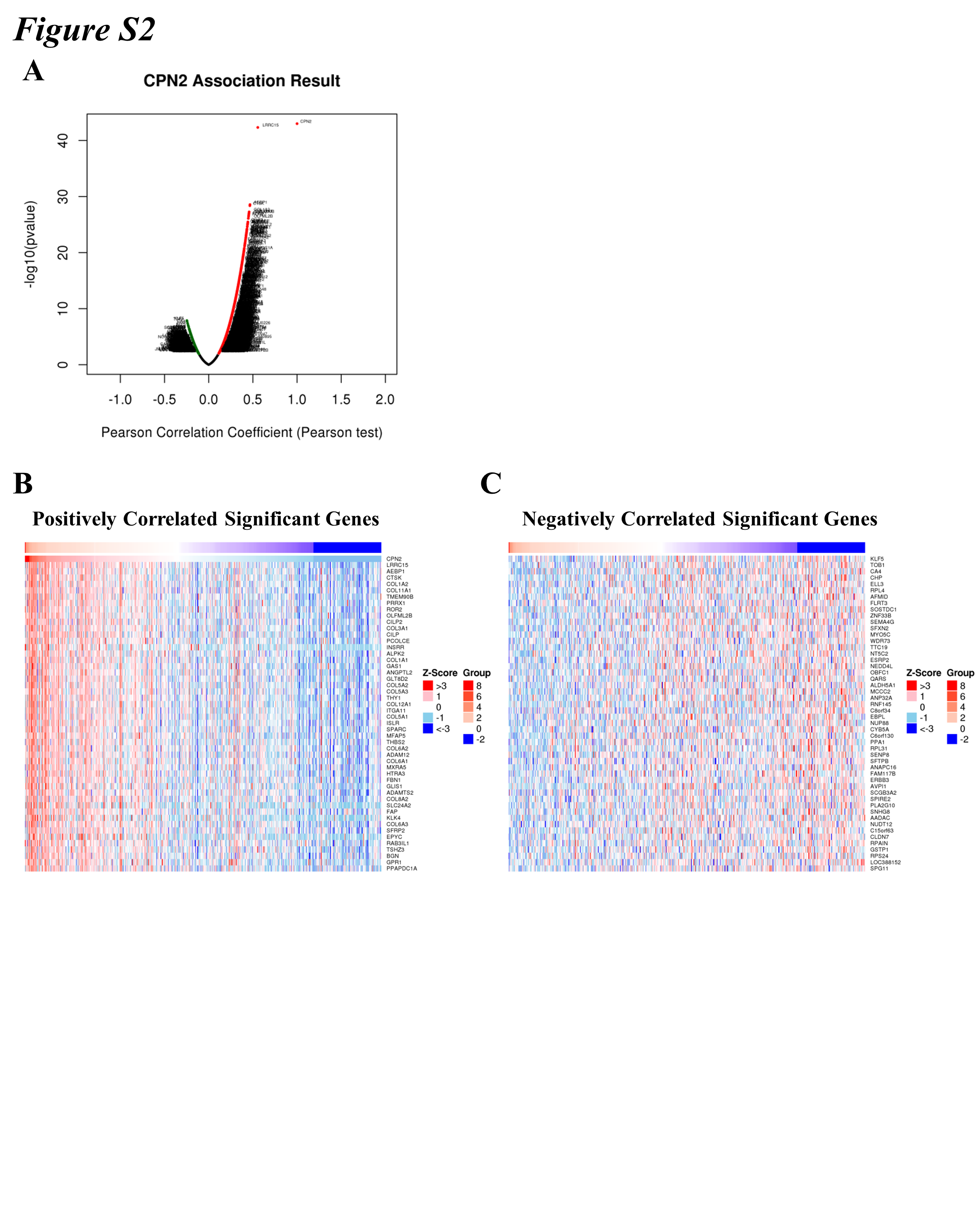

Supplement: Supplementary Figure 2 — CPN2 co-expression genes in lung adenocarcinoma. (A) Volcano plot of CPN2 co-expression genes according to Pearson coefficient. Red dots denote a positive correlation, and blue dots denote a negative correlation. (B) Heat map of the top 50 significantly positively correlated genes with CPN2. (C) Heat map of the top 50 significantly negatively correlated genes with CPN2. [file Image_2.tif]

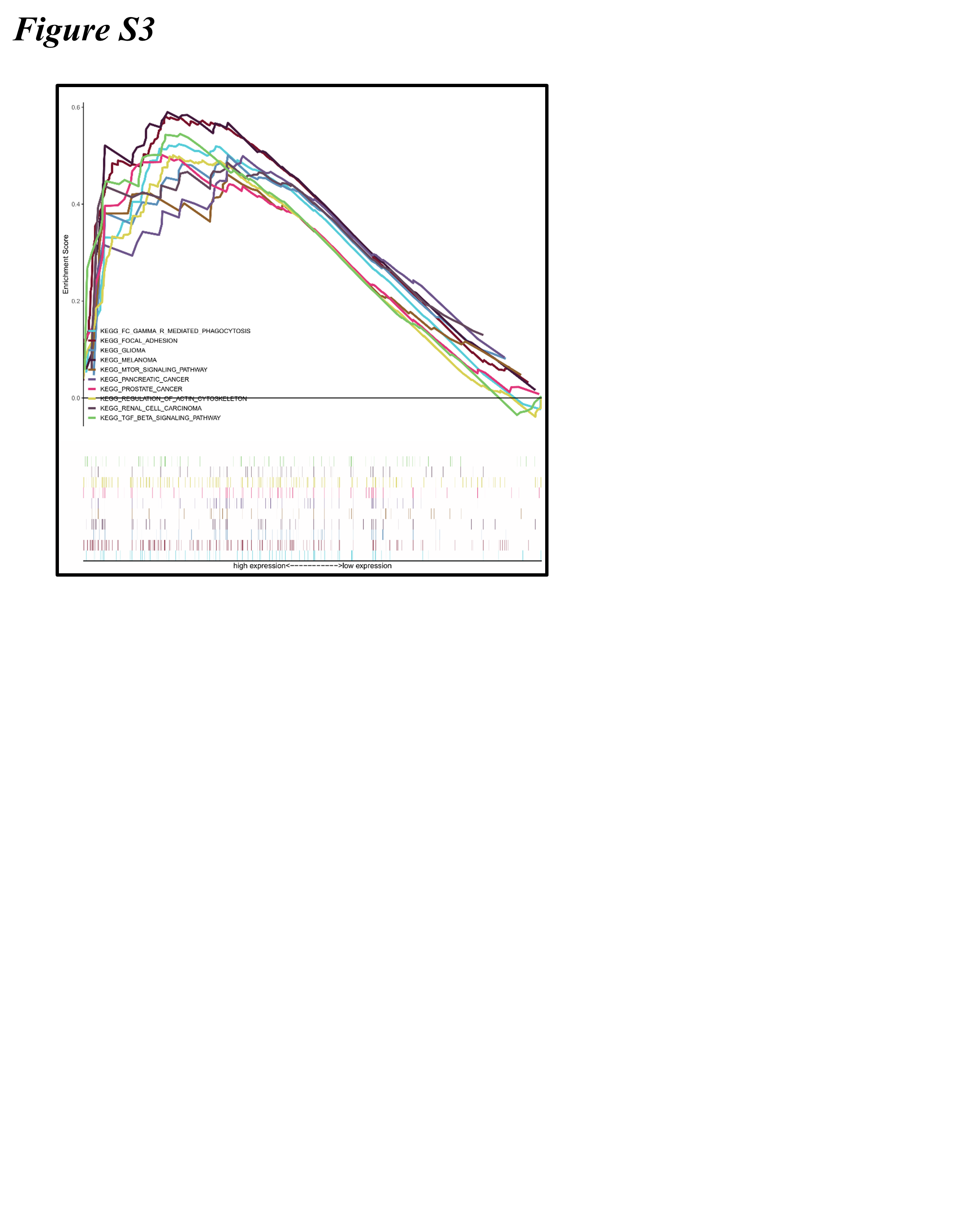

Supplement: Supplementary Figure 3 — Kyoto Encyclopedia of Genes and Genomes enrichment analysis of its interacted genes through Search Tool for the Retrieval of Interacting Genes/Proteins analysis showing the top 10 enrichment pathways according to the normalized enrichment score. False discovery rate <0.05 was considered statistically significant. [file Image_3.tif]

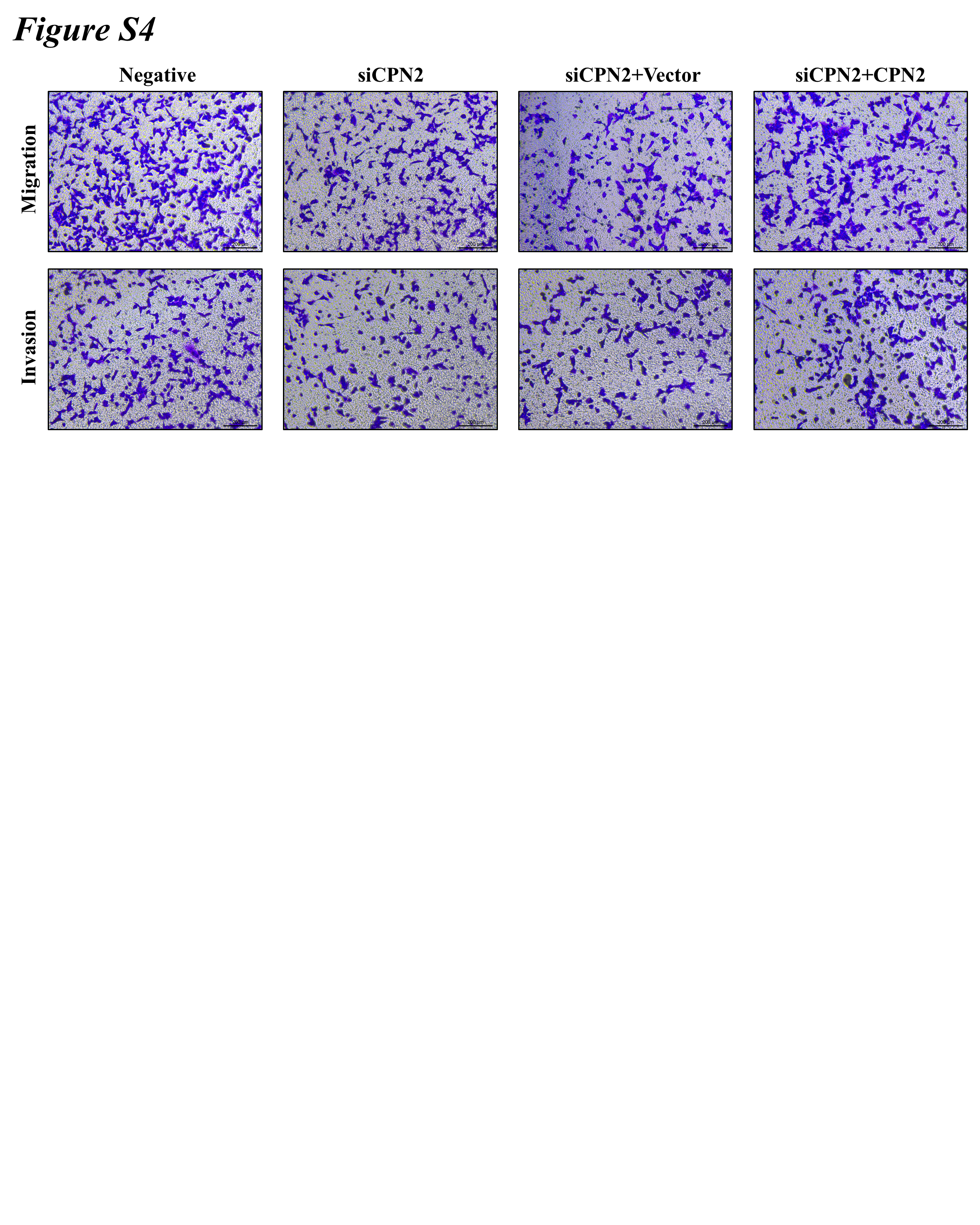

Supplement: Supplementary Figure 4 — CPN2 overexpression significantly promoted the migration and invasion ability in lung cancer cell with CPN2 knockdown by Transwell assay. [file Image_4.tif]
