# Supplementary material for: Carboxypeptidase N2 as a Novel Diagnostic and Prognostic Biomarker for Lung Adenocarcinoma
Source: Front Oncol. 2022 May 23;12:843325. doi: 10.3389/fonc.2022.843325 (PMC9170673; doi:10.3389/fonc.2022.843325)
Supplement: Supplementary Table 1 — Primer sequences used for qRT-PCR. [file Table_1.docx]

**Supplementary Table S1.** Primer sequences used for qRT-PCR.

| Gene | Primer sequence (5’-3’) | Length(bp), position | Annealing temperature(℃) | |
| --- | --- | --- | --- | --- |
| *CPN2* | Forward: GGTGTTCTGCTCAGATGAGGA | 96, | | 60 |
|  | Reverse: AATGTGGTGAACGAGGTCTCC | 96 to 191 | |  |
| *β-actin* | Forward: CCACGAAACTACCTTCAACTCC | 132, | | 60 |
|  | Reverse: GTGATCTCCTTCTGCATCCTGT | 906 to 1037 | |  |
