# Supplementary material for: Carboxypeptidase N2 as a Novel Diagnostic and Prognostic Biomarker for Lung Adenocarcinoma
Source: Front Oncol. 2022 May 23;12:843325. doi: 10.3389/fonc.2022.843325 (PMC9170673; doi:10.3389/fonc.2022.843325)
Supplement: Supplementary Table 2 — Target sequences used for the knockdown of CPN2. [file Table_2.docx]

**Supplementary Table S2.** Target sequences used for knockdown of *CPN2*.

| Gene | Primer sequence (5’-3’) |
| --- | --- |
| *CPN2-1* | Forward: GGAGUGUGCGGCCUAACAATT |
|  | Reverse: UUGUUAGGCCGCACACUCCTT |
| *CPN2-2* | Forward: GCAUAACAUUACAAGACGATT |
|  | Reverse: UCGUCUUGUAAUGUUAUGCTT |
| *CPN2-3* | Forward: CGUGGCCGGACGAAAGCAATT |
|  | Reverse: UUGCUUUCGUCCGGCCACGTT |
| *Control* | Forward: TTCTCCGAACGTGTCACGT |
